# The effects of prenatal psychosocial work stress on adverse pregnancy outcomes: A comprehensive systematic review and meta-analysis

**DOI:** 10.5271/sjweh.4236

**Published:** 2025-09-01

**Authors:** Wubet Taklual Admas, Ai Ni Teoh, Gi Kunchana Chonu

**Affiliations:** 1 James Cook University, School of Social and Health Sciences, Singapore.; 2 Debre Tabor University, College of Health Sciences, Department of Public Health, Ethiopia.

**Keywords:** birth weight, fetal growth, gestational hypertension, occupational hazard, pre-eclampsia, pregnancy loss, preterm

## Abstract

**Objective:**

Psychosocial work stress is a predictor of adverse pregnancy outcomes. However, there is limited comprehensive and conclusive evidence available on the associations between psychosocial work stress and adverse pregnancy outcomes. This systematic review and meta-analysis paper addressed this gap by synthesizing the available evidence.

**Methods:**

Studies were retrieved from six electronic databases that include pregnant mothers as study population, psychosocial work stress as variable exposure, and adverse pregnancy outcomes – including pregnancy loss, gestational hypertension and diabetes mellitus, preterm birth, low birth weight, and low fetal growth – as the outcomes of interest. The quality and certainty of evidence were assessed. Depending on the study characteristics, either a fixed or random effect model was employed. Heterogeneity was assessed using I^2^ statistics, and further subgroup and sensitivity analysis was employed as appropriate.

**Results:**

A total of 26 studies (N=1 346 686) were included. Psychosocial work stress decreased birth weight by 77.09 grams, increased the occurrence of preeclampsia by 50%, and preterm birth by 18% with moderate certainty of evidence, and increased the chance of pregnancy loss by 20% with low certainty of evidence. With a low grading scale, low birth weight and small-for-gestational-age had no significant association with psychosocial work stress.

**Conclusions:**

Psychosocial work-stress increased the risks of pre-eclampsia, preterm birth, and pregnancy loss, and decreased fetus weight. Therefore, occupational therapists, employers, policy makers, and relevant stakeholders should work together to minimize the impact of psychosocial work-stress on the mother and baby.

Adverse pregnancy outcomes are unfavorable physiologic outcomes that occur during pregnancy, labor, and post-natal period on the mother and her fetus (1). These include pregnancy loss (miscarriage and stillbirth), preterm birth (PTB), low birth weight (LBW), small-for-gestational-age (SGA), hypertensive disorder of pregnancy (HDP), and gestational diabetes mellitus (GDM) (2). Adverse pregnancy outcomes are the leading causes of maternal and child morbidity and mortality (1). It contributed more than a third of maternal mortality (1) and three-fourths of neonatal mortality (3). Research shows that these outcomes are higher in Southeast Asia and Sub-Saharan African countries (1, 4).

Mothers who experience adverse pregnancy outcomes are at higher risks of developing psychosocial health problems, cardiovascular diseases, diabetes mellitus, and kidney disease later in life (5–8), while their surviving child is more likely to have neurodevelopmental disorders that may last into adulthood (9–13). Therefore, addressing and identifying the predictors of these unfavorable outcomes is critical to promoting mothers’ and their children’s short-term and long-term health.

Evidence has shown that work stress is one of the predictors of adverse pregnancy outcomes (14–19), which leads to different acute and chronic health problems (20–23). Work stress includes physical (eg, heavy physical work-load, whole-body vibration, etc.) (18, 24) and environmental stress (eg, noise-exposure, heat-exposure, etc.) (19, 25) in workplace settings and stress arises from high job demands, low decisional latitude, limited social and organizational support, job insecurity, role ambiguity, organizational imbalance, bullying, and inadequate rewards, which are collectively referred to as psychosocial work stress (PSWS) (20, 21, 23). The physical or environmental aspects of work stress have been investigated in the previous systematic reviews and are linked to adverse pregnancy outcomes (18, 19, 24, 26, 27). However, the psychosocial aspect of work stress (PSWS) is rarely studied.

More than 90% of pregnant women continue working during pregnancy, and many of them work until delivery due to financial necessity and job demand (28). Often, they work under similar conditions to those of non-pregnant workers (29, 30). In many countries, there is no legal protection for pregnant mothers in the workplace, and employers fail to offer special accommodations such as lighter workloads or flexible schedules (31, 32).

Although 47.4% of employed individuals are women in the global labor market (33), they are more prone to PSWS and its detrimental effects, primarily because they are more vulnerable to work-life and work-family imbalance, role overload including a second shift at home, and vocational strain (34, 35). Physiological stress arising from physical and hormonal changes associated with pregnancy may further aggravate the effect of PSWS on pregnant mothers (36).

Studies investigating the effects of PSWS on pregnancy outcomes report inconsistent findings, making it difficult to draw firm conclusions. While some studies showed that PSWS contributes to adverse pregnancy outcomes, including HDP (17, 37), pregnancy loss (14), PTB (38–40), LBW (14, 15, 41), and SGA (16, 42), other studies have found no significant associations (43–50). The inconsistent findings were mainly due to variations in study design, sample sizes, types of stressors, and duration of exposure. They highlight the necessity to consolidate the available evidence to understand the impact of PSWS on pregnancy outcomes.

Therefore, this systematic review and meta-analysis paper aimed to fill this gap by consolidating the available evidence on the associations between PSWS and adverse pregnancy outcomes, including HDP, pre-eclampsia, GDM, pregnancy loss, PTB, LBW, and SGA. The results are crucial for advising employers, occupational therapists, and clinicians, and making policy recommendations for relevant stakeholders. The overall impacts are crucial for achieving Sustainable Development Goal (SDG) 3 in reducing maternal mortality to<70 per 100 000 live births and neonatal mortality to <12 per 1000 live births by the end of 2030 (51).

## Methods

The systematic review and meta-analysis was performed following the updated Preferred Reporting Items for Systematic Review and Meta-analysis (PRISMA) guideline reporting (52). The review protocol was registered in PROSPERO (CRD42024592293).

### Search strategy

A comprehensive literature search was employed using six electronic databases including SCOPUS, PsycINFO, CINAHL, PubMed, Cochrane Library, and Science Citation Index Expanded without restricting countries and publication years. To ensure adequate coverage, we used Google Scholar for grey literature search and additional search through the reference lists of the studies. A broad range of keywords, controlled vocabulary terms, and Medical Subject Headings for each database were used. Boolean operators (OR, AND, NOT) were applied for precision and accuracy (see supplementary material, www.sjweh.fi/article/4236, file 1).

### Inclusion and exclusion criteria

We selected research studies that examined PSWS and adverse pregnancy outcomes based on the PICO framework (53): (i) Population (singleton pregnant mothers engaged in paid employment); (ii) Intervention (PSWS); (iii) Comparator (singleton pregnant mothers in paid employment without or with low PSWS); and (iv) Outcomes (HDP, pre-eclampsia, GDM, pregnancy loss, LBW, PTB, and SGA). In this review, studies conducted using both observational and interventional designs were included. Case reports, case series, conference proceedings, reviews, editorials, commentaries, government reports, non-human studies, and studies published in languages other than English were excluded. In addition, studies on pregnant mothers exposed to other nonwork-related psychosocial stress, physical workload, or other environmental factors were excluded, if psychosocial work factors were not specifically identified as exposure variables as described below.

### Exposure

PSWS is the exposure variable, measured using different models in the studies reviewed in this paper. These include the Job Demand–Control (JDC) model, the Job Demand–Resource (JDR) model, and the Effort–Reward Imbalance (ERI) model. Karasek (54) developed and validated the JDC model. According to this model, job demand is defined as emotional and cognitive load, including tight deadlines, prolonged working hours, and task complexity. Job control refers to decision-making authority, task management, skill utilization without external influence, and the presence of flexible working conditions. The model identifies four job types: low strain job (low job demand with high control), active job (high job demand with high control), passive job (low job demand with low control), and high strain job (high job demand with low control) (54).

Bakker & Demerouti (55) developed the JDR model. According to the model, job resources include support from supervisors and colleagues, team cohesion, teamwork, skill development opportunities, and access to training programs. According to the JDR model, individuals facing high job demands and low job resources are considered experiencing high job strain (55).

Siegrist (56) proposed the ERI model, in which work effort refers to the demands placed on an employee, while reward encompasses the employee’s expectations for compensation in the form of rewards, promotion, or job security. When an employee expends high effort but receives low reward, it results in job stress (56).

### Outcome variables

In this review, seven key outcomes were included: HDP, pre-eclampsia, GDM, pregnancy loss, PTB, LBW, and SGA. To ensure consistency across studies, standard operational definitions were applied to each outcome: (i) *Hypertensive disorder of pregnancy (HDP) –* blood pressure ≥140/90 mmHg after 20 weeks of gestation without proteinuria (57); (ii) *Pre-eclampsia* – high blood pressure (≥140/90 mmHg) after 20 weeks of gestation and with either proteinuria or a sign of organ dysfunction (57); (iii) *Gestational diabetes mellitus (GDM)* – Development of glucose intolerance after 20 weeks; glucose level ≥92 mg/dL (5.1 mmol/L) in fasting or one hour after a meal ≥180 mg/dL (10.0 mmol/L) or two hours after a meal ≥153 mg/dL (8.5 mmol/L) (58); (iv) *Pregnancy loss* – A combination of miscarriage and stillbirth are considered pregnancy loss in this review; (v) *Miscarriage (spontaneous abortion)* – Pregnancy ended with ≤20 weeks of gestation (59); (vi) *Stillbirth/fetal loss*: Fetal death that occurs after 20 weeks of gestation; (vii) *Low birth weight (LBW)* – Birth weight lower than 2500 grams (60); *Small-for-gestational-age (SGA) –* Birth weight below 10^th^ percentile for gestational age based on sex; (viii) *Preterm birth (PTB)* – Giving birth before 37 weeks of gestation (60).

### Study selection

**Figure 1:**
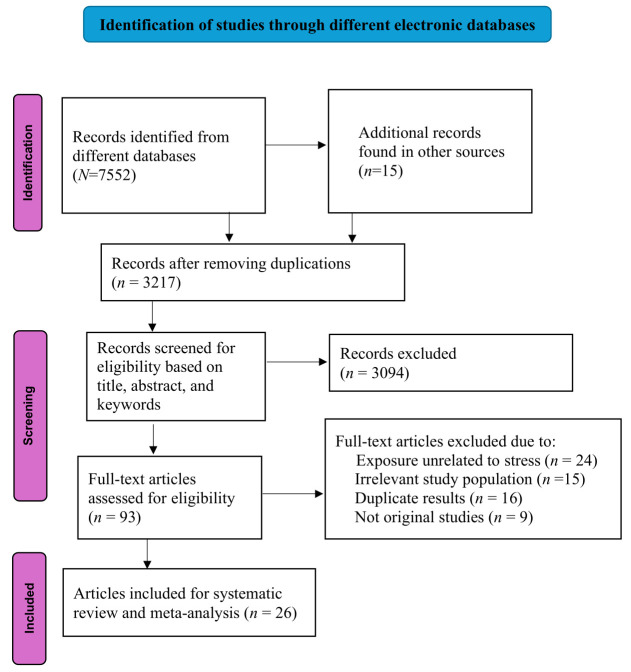
PRISMA flow diagram for studies selection process.

All articles retrieved from electronic databases were exported to EndNote 21 for duplication removal. Two authors conducted initial titles and abstract screening using a structured checklist. Studies that met the selection criteria by either reviewer were eligible for full-text screening. The same reviewers did full-text screening following the inclusion and exclusion criteria. Any discrepancies between the reviewers were resolved through discussions and, in cases of irreconcilable differences, a third reviewer made the final decision. The study selection process was presented in the PRISMA diagram (see figure 1).

### Data extraction

After developing a structured data abstraction format, a pilot test was conducted on a few randomly selected articles to ensure that the form included all relevant information to answer the review question and was user-friendly. Two authors extracted the data independently, and discrepancies were resolved through discussions. Extracted data included study characteristics (authors, years of publications, countries, study designs, and period), participant characteristics (study population and sample sizes), exposure variables (exposure variable measurement, durations of exposure, and proportions of exposed participants), outcomes (HDP, pre-eclampsia, GDM, pregnancy loss, PTB, birthweight, and SGA), results (proportions of outcomes of interest, effect estimates, controlling confounders), and other important information (see table 1).

**Table 1:** Details of the included studies in the review. [AHR=adjusted hazard ratio; AOR=adjusted odds ratio; ARR=adjusted relative risk; BP=blood pressure, CI=confidence interval; COR=crude odds ratio; ERI=effort–reward imbalance; GDM=gestational diabetes mellitus; HDP=hypertensive disorder of pregnancy; JDC=job demand–control; LBW=low birth weight; LSS=low social support; NA=not applicable; P=proportion; PTB=preterm birth; SE=standard error; SGA=small- for-gestational-age; SS=social support; Wks=wks]

Author (year), country	Study design & period	Study population	Exposure variable & measurement	Time of exposure measurement & duration of exposure	Sample size & women exposed to high job strain (P)	Outcomes of interest & outcome measurement	Women who develop outcomes of interest (P)	Main finding (95% CI)
Brandt et al (1992) (14), Denmark	Case–control. 1983–1985	Commercial and clerical female workers	Job strain e valuated by the JDC model using Karasek’s scale	After childbirth with a recall period of 2.5–4.5 years, exposed any time during pregnancy	N=2490, 31.16%	Spontaneous abortion before 28 wks of gestation	45.18%	High job strain AOR=1.28 (1.05–1.57)
					N=1786 29.05%	Congenital malformation	23.57%	High job strain AOR=1.23 (0.93–1.63)
					N=1727 29.76%	PTB=delivery before 36 wks of gestation	20.96%	High job strain AOR=1.03 (0.77–1.39)
					N=1712 30.54%	LBW=birth weight is less than 2500 g	20.26%	High job strain AOR=1.46 (1.05–2.04)
					N=1980 29.54%	SGA=birthweight in the lowest 5^th^ percentile	31.06%	High job strain AOR=1.08 (0.83–1.40)
					N=1493 29.7	Still birth and death in the 1^st^ year of life	8.57%	High job strain AOR=1.42 (0.90–2.24)
Breet et al (1997) (38), USA	Case–control, 1988–1991	African American and Caucasian	Job strain evaluated by the JDC model using Karasek’s scale	After 6 months of childbirth. Duration of exposure=>30 wks & 4–29 wks	N=398 35.43%	PTB=give birth before 37 completed wks	36.18%	High strain job among >30 wks of exposure (AOR=1.8 (1.1–3.1) and high strain job among < 30 wks of exposure (AOR=1.0 (0.5–2.0)
Ceron-Mireles et al (1996) (72), Mexico	Cross-sectional, 1992	General population	Job strain evaluated by the JDC model using Karasek’s scale	Immediately afterchildbirth. Exposed ≥ 3 months during pregnancy	N=2320 22.76%	PTB=Give birth before 37 completed wks	11.81%	High job strain COR=1.16 (0.90–1.50)
					N=2309 22.74%	SGA=below the 10th percentile of weight for a given GA	10.91%	High job strain COR=1.23 (0.95–1.60)
Croteau et al (2007) (39), Canada	Case–control, 1997–1999	General population	Job strain evaluated by the JDC model using Karasek’s scale	Within 32 days of childbirth	N=962 (within 24 wks of gestation) 41.68%	PTB=Give birth before 37 completed wks	15.38%	High job strain with minimal social support within 24 wks of pregnancy only AOR=1.0 (0.7–1.3)
					N=483 (late 24 wks of gestation) 33.75%	PTB=Give birth before 37 completed wks	13.87%	High job strain with minimal social support late 24 wks of pregnancy AOR=1.2 (0.8–1.8)
					N=2627 (throughout pregnancy 25.96%	PTB=Give birth before 37 completed wks	16.94%	High job strain with minimal social support throughout pregnancy AOR=1.4 (1.1–1.8)
Escriba-Agüir et al (2001) (71), Spain	Case–control, 1994–1995	General population	Job demand, evaluated using the job demand model	Within two days after delivery	N=572 20.98%	PTB=birth before 37 wks of gestation	39.86%	High strain job AOR=1.4 (0.95–2.26)
Fenster et al (1995) (43), USA	Prospective, 1990–1991	General population	Job strain evaluated by the JDC model using the Karasek’s scale and social support	Within 13 wks of conception	N=3953 8.4%	Miscarriage= pregnancy ended ≤20 wks of gestation	9.9%	High job strain AOR=1.18 (0.81–1.71). Low social support AOR=0.91 (0.72–1.16)
Haelterman et al (2007) (70), Canada	Case–control 1997–1999	General population	Job strain evaluated by the JDC model using Karasek’s scale and social support	Within 30 days of delivery	N=4538 23.94%	Preeclampsia=BP ≥140/90 mmHg with albuminuria.	2.25%	High job strain AOR=1.7 (0.8–3.3)
						HDP=BP ≥140/90 mmHg without albuminuria	2.23%	High job strain AOR=1.0 (0.6–1.9)
Henriksen et al (1994) (44), Denmark	Prospective 1989–1991	General population	Job strain evaluated by the JDC model using Karasek’s scale	16^th^ and 30^th^ wks of gestation	N=3407 24.89%	SGA=birthweight below the 10^th^ percentile	9.1%	High job strain AOR=1.1(0.7–1.6)
						PTB=give birth before 37 complete wks	3.96%	High strain job AOR=1.3(0.7–2.2)
Homer et al (1990) (15), USA	Prospective, 1979–1983	General population	Job strain evaluated by the JDC model using Karasek’s scale	9wks before pregnancy and during pregnancy	N=780 33.6%	LBW=birthweight less than 2500 grams	6.25%	High job strain with low motivation to work RR=8.0 (1.3–37)
						PTB=Give birth before 38 completed wks	3.4%	High job strain with low motivation to work RR=8.4 (1.4–50.2)
Author (year), country	Study design & period	Study population	Exposure variable & measurement	Time of exposure measurement & duration of exposure	Sample size & women exposed to high job strain (P)	Outcomes of interest & outcome measurement	Women who develop outcomes of interest (P)	Main finding (95% CI)
Klonoff-Cohen et al (1996) (65), USA	Case– control, 1987	Nulliparous women	Job strain evaluated by the JDC model using Karasek’s scale	1 year after pregnancy	N=123 30.89%	Preeclampsia=BP ≥140/90 mmHg with proteinuria	56.1%	High job strain compared with other workers AOR=2.1 (0.7–6.2). High job strain compared with non–workers AOR=3.1 (1.2–7.8)
Landsbergis et al (1996) (66), USA	Prospective 1987–1989	General population	Job strain evaluated by the JDC model using Karasek’s scale	13, 28, & 36 wks of gestation	N=575 5%	Preeclampsia=BP ≥140/90 mmHg with proteinuria	1.9%	High job strain AOR=1.6 (0.3–10.3)
						HDP=BP ≥140/90 mmHg without albuminuria	2.78	High job strain AOR=1.7 (0.3–8.9)
Larsen et al (2013) (45), Denmark	Prospective, not mentioned	General population	Job strain evaluated by the JDC model using Karasek’s scale	1^st^ or 2^nd^ trimester of pregnancy	N=48 890 6.83%	PTB=give birth before 37 completed wks	4.86%	High job strain AOR=0.98 (0.82 –1.16). High job strain with low social support AOR=1.39 (0.86 –2.23)
						SGA=birthweight below the 10^th^ percentile	9%	High job strain AOR=1.01 (0.89 – 1.14) High job strain with low social support AOR=1.06 (0.73–1.53)
Larsen et al (2014) (69), Denmark	Prospective-Not stated	General population	Job strain evaluated by the JDC model using Karasek’s scale	1^st^ or 2^nd^ trimester of pregnancy	N=60 386 6.7%	All congenital malformations	5.1%	High job strain AOR=0.99 (0.85–1.15)
Lee et al (2011) (41), Korea	Prospective, not stated	General population	Job strain evaluated by the JDC model using Karasek’s scale and ERI model	1^st^ trimester	N=310 NA	LBW measured in continues variables	NA	High job strain using JDC isn’t significantly associated (b(SE)=–33.39 (61.03). ERI doesn’t affect birth weight after adjustment
Lissåker et al (2022) (46), Sweden	Prospective, 1994–2014	General population	Job strain evaluated by job exposure matrix using Karasek’s scale and JD-R (the information collected on job specific).	Starting 1^st^ trimester	N=1 080 850 24.2% (JDC) 23.3% (LSS)	Preeclampsia=BP ≥140/90 mmHg with proteinuria	3% 2.71% (LSS)	High job strain with ARR=1.02 (0.98–1.06) Low social support ARR=0.92 (0.89–0.96)
						HDP=BP ≥140/90 mmHg without albuminuria	3.97% 3.67% (LSS)	High job strain with ARR=1.02 (0.99–1.05) Low social support ARR=0.93 (0.90–0.96)
						GDM: Fasting glucose ≥ 92 mg/dl	0.8% 0.7% (LSS)	High job strain with ARR=1.0 (0.94–1.07) Low social support ARR=0.91 (0.85–0.98)
Marcoux et al (1999) (17), Canada	Case–control, 1986	Primi-gravida women	Job strain evaluated by the JDC model using Karasek’s scale	Within one month of delivery	N=730 20.27%	Preeclampsia=BP ≥140/90 mmHg with proteinuria	17.53%	High job strain AOR=2.1 (1.1–4.1)
						HDP=BP ≥140/90 mmHg without albuminuria	27.53%	High job strain AOR=1.3 (0.8–2.2)
Meyer et al (2016) (37), USA	Prospective, study period not stated	Health care workers	Job strain evaluated by the JDC model using Karasek’s scale and ERI	Repeated measurement in each trimester	N=61 14% (ERI) 21% (JDC)	HDP=NA (only systolic BP assessed)	NA	High ERI B(SE)=8.8 (2.7) P=0.001 High job strain B(SE)=3.3(2.3) P=0.1
Meyer et al (2017) (67), USA	Prospective, study period not stated	Health care workers	Job strain evaluated by the JDC model using Karasek’s scale and ERI	Repeated measurement in each trimester	N=55 14% (ERI) 21% (JDC)	Birth weight measure continues	NA	High ERI B(SE)= 317(280g), P=0.26 High job strain B(SE)=7(167g), P=0.87
Meyer et al (2007) (40), USA	Cross-sectional, 2000	General Population	Job strain evaluated by the JDC model using Karasek’s scale	Any time during pregnancy	N=26,408 19.34%	LBW=birth weight <2500 g	5.5%	High job strain AOR=1.11 (0.92–1.34)
						PTB=delivery before 37 wks gestation	8.3%	High job strain AOR=1.17 (1.00–1.36)
Oths, et al (2001) (68), USA	Prospective, 1993–1996	General population	Job strain evaluated by the JDC model using Karasek’s scale	14 and 28 wks of gestation	N=480 36%	Birth weight (continuous in grams)	NA	Work in high job strain has unadjusted difference in Birth weight 190g (95%CI 48-333)
Sejbaek et al (2018) (47), Denmark	Prospective, 1996–2002	General population	Job strain evaluated by the JDC model using Karasek’s scale	During the first or second trimester pregnancy	N=47582 8.2%	SGA=birthweight below the 10^th^ percentile	8.1%	High job strain AOR=1.01 (0.90–1.13)
						LGA=birthweight above the 90^th^ percentile	12.43%	High job strain AOR=1.16 (1.07–1.26)
Author (year), country	Study design & period	Study population	Exposure variable & measurement	Time of exposure measurement & duration of exposure	Sample size & women exposed to high job strain (P)	Outcomes of interest & outcome measurement	Women who develop outcomes of interest (P)	Main finding (95% CI)
Tuntiseranee et al (1998) (16), Thailand	Prospective, 1994–1995	General population	Job strain evaluated by the JDC model using Karasek’s scale	At 17 and 32 wks of gestation	N=1797 NA	PTB=delivery before 37 wks gestation	4.9%,	High job strain AOR=0.7(0.2–2.3)
						SGA=birthweight below the 10^th^ percentile	2.7%,	High job strain AOR=12.7(3.1–51.8)
Vollebregt et al (2007) (48), Netherland	Prospective, 2003–2004	Nulliparous	Job strain evaluated by the JDC model using Karasek’s scale	Before 24 wks of gestation	N=3679 5.65%	Preeclampsia=BP ≥140/90 mmHg with proteinuria	3.5%	High job strain AOR=1.61 (0.75–3.49)
						HDP=BP ≥140/90 mmHg without albuminuria	4.4%	High job strain AOR=1.03 (0.48–2.20)
Vrijkotte et al (2021) (49), Netherland	Prospective, 2003–2004	General population	Job strain evaluated by the JDC model using Karasek’s scale	Before 24 wks of gestation	N=4865 6.7%	PTB=delivery before 37 wks gestation	5.2%	High job strain AOR=1.02 (0.60–1.76)
Vrijkotte et al (2009) (42), Netherland	Prospective, 2003–2004	General population	Job strain evaluated by the JDC model using Karasek’s scale	Before 24 wks of gestation	N=7096 P=4.2%	Birth weight (continuous in grams	NA	High job strain B(SE)=–72 (26), P value=<0.01
						SGA=below the 10^th^ percentile for gestational age	NA	High job strain AOR=1.5 (1.1–2.1)
Zhu, et al (2004) (50), Denmark	Prospective, 1998–2001	General population	Job strain evaluated by the JDC model using Karasek’s scale	Before 25 wks of gestation	N=41 769 P=7.76%	Miscarriage=A fetal loss before 28 wks	1.15%	High job strain AHR=1.00 (0.68 –1.46)
						Stillbirth=fetal death ≥28 wks	0.32%	High job strain AHR=0.85 (0.40 –1.82)

### Study quality assessment (risk of bias)

Methodological quality of the included studies was assessed using the Joanna Briggs Institute (JBI) evaluation tool (61). The JBI checklist consists of several domains specific to the study design. It includes 13 criteria for cohort studies, 10 for case–control studies, and 8 for cross-sectional studies. No trials or interventional studies were found among the included studies. According to the JBI checklist, studies were categorized into three levels of risk based on the percentage of criteria marked as “yes.” Those scoring ≥70% were considered low risk, 50–69% medium risk, and <50% high risk (62). Two independent reviewers evaluated each study, and any differences were resolved through discussions (see supplementary file 2).

### Certainty of evidence assessment

We used the Grade Recommendations Assessment Development and Evaluation (GRADE) system to evaluate the quality of the evidence into four levels of certainty: high, moderate, low, and very low. Initial ratings start at “high” for randomized controlled trials and “low” for observational studies. The quality of evidence can be downgraded or upgraded based on eight specific factors. Factors that downgrade the ratings are risk of bias, inconsistent results, indirectness, imprecision, and publication bias. On the other hand, factors that upgrade the grading score are the magnitude of effect size, residual plausible confounders, and dose-response (63, 64).

When rating the GRADE factors, the risk of bias is deemed serious if more than half of the studies are rated a high risk of bias. Indirectness is a serious concern if over half of the studies show substantial deviations from the PICO framework. Inconsistency is classified as serious if the *I^2^* statistics are >50%. Publication bias is a concern if there is asymmetry in the funnel plot or if the Egger test yields statistically significant results. Imprecision is categorized as serious if the total sample size of the included studies does not meet the Optimal Information Size criteria, or if the confidence intervals (CI) contain null values (24). The quality of the evidence is upgraded in the presence of large magnitude effects (risk ratio >2 or <0.5), limiting a plausible residual bias, or a dose–response relationship (24).

### Data analysis

The extracted data from the Excel sheet was imported into STATA version 18 for further analyses, regardless of the sample size. Odds ratios (OR) were used as effect sizes. The natural logarithm of the OR along with their 95% CI and corresponding standard errors were calculated for the meta-analysis. Dichotomous and continuous variables were analyzed separately. When heterogeneity was low or absent, study characteristics were similar in qualitative assessments, and there were five or more studies, we employed a fixed effects model with the inverse-variance method. In other cases, we used a random effects model with the restricted maximum-likelihood approach. Variability between the studies was evaluated using *I^2^* statistics and Q-test. Based on *I^2^* statistics, heterogeneity is defined as low (0–24%), medium (25–75%), or high (>75%). If the *I^2^* statistic was >50%, further subgroup analysis and sensitivity analyses were employed. Publication bias was assessed using the regression-based Egger test, and if detected, a non-parametric trim-and-fill analysis was employed. Statistical significance was defined at P<0.05.

## Results

### Search results

We retrieved 7552 articles from six electronic databases and 15 articles from the Google search engine. After removing duplicates, we screened 3217 articles based on their titles and abstracts for eligibility. After excluding ineligible articles based on the PICO framework, 93 articles were included for full-text screening. Of these, 67 studies were excluded for reasons specified in figure 1. Finally, 26 articles met the criteria and were included in the systematic review and meta-analysis.

### Study characteristics

This systematic review and meta-analysis included 26 studies without time period restriction from 9 countries: 9 studies were based in USA (15, 37, 38, 40, 43, 65–68), 6 in Denmark (14, 44, 45, 47, 50, 69), 3 in Canada (17, 39, 70), 3 in the Netherlands (42, 48, 49), and 1 each in Spain (71), Sweden (46), Mexico (72), Korea (41), and Thailand (16). All included studies used an observational study design: 18 prospective cohort studies (15, 16, 37, 41–50, 65–69), 6 case–control (14, 17, 38, 39, 70, 71), and 2 cross-sectional study design (40, 72). These studies involved a total of 1 368 127 pregnant mothers with sample sizes ranging from 67 to 1 102 230. Regarding participant characteristics, 21 studies recruited pregnant women from the general population, 2 studies focused specifically on healthcare workers (37, 67), 1 on primigravid women (17), 1 on nulliparous women (65), and 1 on female commercial and clerical workers (14) (see table 1). Moreover, all studies controlled the effects of confounding variables.

All studies used PSWS as an exposure variable and evaluated it using the JDC model. However, 2 studies used the ERI model (37, 67), and 4 used the JDR model (39, 43, 46, 70) as an additional PSWS assessment method (39, 43, 46, 70). In 17 studies, exposure time was measured as experiencing PSWS from the first trimester of pregnancy (15, 16, 37, 42–50, 66–69), and the rest were measured as experiencing PSWS at any time during pregnancy. Only 1 study assessed preconception stress before 9 weeks of pregnancy (15). The proportion of pregnant mothers exposed to high PSWS was 4.2–36% across studies.

### Risk of bias and certainty of evidence assessment

Quality assessment showed that 18 studies had a low risk of bias (14, 15, 38–40, 42–44, 47–50, 65, 66, 68, 71, 72), while 8 exhibited a medium risk of bias (14, 16, 37, 41, 45, 46, 67, 69, 70). Common sources of bias in cohort studies were differences between study groups and populations (15, 16, 39–45, 47–50, 66, 68–72), failure to address incomplete follow-ups (16, 37, 41, 45, 50, 67, 69), a lack of explanation for incomplete follow-ups (15, 16, 41, 44, 69), use of inappropriate statistical analysis (16, 37, 41, 67, 68), and invalid and unreliable measurement of outcomes (37, 41, 67). The risks of bias found in case–control studies were non-matching groups other than the presence of outcomes of interest (17, 38, 39, 70, 71) and inconsistent criteria for identifying cases and controls (70, 71). In cross-sectional studies, the primary source of bias was the absence of specific criteria for selecting study participants (40) (see supplementary file 2).

The GRADE assessment was conducted for seven pregnancy outcomes, with evidence ratings ranging from very low to medium. All the studies included were observational, starting with a baseline of a low rate. Two outcomes were downgraded to very low; three outcomes were upgraded to medium, and two outcomes remained unchanged. Downgrading was due to inconsistency (N=2), imprecision (N=1), and publication bias (N=2). The upgrade was driven by a large effect size and contracting residual confounders (see supplementary file 3)

### Maternal complications

*Gestational hypertension.* Six studies examined the association between PSWS and HDP (N=1 111 813) (17, 37, 46, 48, 66, 70). However, one study was excluded from the meta-analysis due to differences in the exposure variable measurement (37). Although an outlier was observed in the qualitative assessment and the forest plot among the included studies, there was no evidence of publication bias detected (β*_1_*=0.4, P=0.45). In addition, retaining the outlier did not significantly alter the pooled effect, and therefore it was included in the final analysis (46) (see supplementary figure S1). With a low certainty of evidence, the final pooled effect showed no significant relationship between high PSWS and HDP [OR 1.02 (95% CI 0.99–1.05)].

*Pre-eclampsia.* Six studies examined the relation between PSWS and pre-eclampsia (N=1 111 936) (17, 46, 48, 65, 66, 70). Among the studies, one study accounted for 99.3% of the total weight, significantly influencing the overall effect size during the sensitivity analysis (46). Due to its substantial impact on the pooled estimate and identified methodological issues, this study was excluded from the final meta-analysis. A subsequent analysis was conducted using the remaining five studies (17, 48, 65, 66, 70). With a medium quality of evidence, the analysis revealed the significant association between PSWS and pre-eclampsia [OR 1.5 (95% CI 1.06–2.13)]. No publication bias was detected (β*_1_*=0.93, P=0.49) (see supplementary figure S2).

*Gestational diabetes mellitus.* A single prospective study conducted in Sweden among the general population (N=1 102 230) reported an exposure rate of 24.2%, using both the JDC and JDR models (46). The prevalence of GDM was 0.8% in the JDC model and 0.7% in the JDR model. After adjusting for confounding variables, PSWS measured using the JDC model had no significant association with GDM [ARR=1 (95% CI 0.94–1.07)]. PSWS measured using the JDR model showed a protective effect [ARR=0.91 (95% CI 0.85–0.98)].

### Obstetric outcomes

*Pregnancy loss.* Three studies (N=48 212) examined the association between PSWS and pregnancy loss (14, 43, 50). With a low certainty of evidence, the pooled effect showed the significant association between high PSWS and pregnancy loss [OR 1.20 (95% CI 1.04–1.40)] (see supplementary figure S3). Both qualitative and statistical assessments indicated no evidence of publication bias (β*_1_*= -1.65, *P*=0.17).

*Preterm birth.* Eleven studies (N=91 791) examined the relationship between PSWS and PTB (14–16, 38–40, 44, 45, 49, 71, 72). However, two studies were excluded from the meta-analysis due to differing operational definitions of the outcome (14, 15). The remaining nine studies (N=89 284) were included in the meta-analysis. With a medium certainty of evidence, the pooled effect indicated that high PSWS has a significant association with PTB [OR 1.18 (95% CI 1.05–1.34)] (see supplementary figure S4). No evidence of publication bias was identified (β*_1_*=0.41, *P*=0.58).

Further analyses were conducted based on study designs and exposure durations. Based on the study designs, the pooled effects of cross-sectional (40, 72), case–control (14, 38, 39, 71), and prospective (15, 16, 44, 45, 49) studies were [OR 1.17 (95% CI 1.02–1.33)], [OR 1.45 (95% CI 1.19–1.77)], and [OR 1 (95% CI 0.85–1.17)], respectively (see supplementary figure S5). Additional sub-group analyses based on the exposure durations of PSWS during pregnancy were also performed; comparing exposure at any time during pregnancy [OR 1.19 (95% CI 1.02–1.33)] to exposure throughout pregnancy [OR 1.20 (95% CI 0.96–1.50)] (see supplementary figure S6).

### Fetal outcomes

*Birth weight.* Seven studies examined the link between PSWS and birth weight. However, their data contains both dichotomous and continuous outcomes. Hence, the studies with categorical and continuous outcomes were analyzed separately.

Firstly, three studies with dichotomous outcomes were included in an analysis (N=28 900) (14, 15, 40). The pooled effect showed non-significant association between PSWS and LBW [OR 2.30 (95% CI 0.70–7.60)], with a very low certainty of evidence (see supplementary figure S7). In addition, a significant publication bias (β*_1_*=11.87, P=0.002) and heterogeneity (*I^2^*=97.5%) were detected. This is because of the small number of studies, and variations in study designs and sample sizes. The non-parametric trim-and-fill analysis did not identify any imputed studies, indicating potential bias. Although the outlier was observed in the forest plot (15), a sensitivity analysis was not performed due to the small number of studies.

Secondly, four studies with the continuous birth weight variable were included (N=7941). All of which employed a prospective cohort study design. With medium certainty of evidence, the pooled effect showed that a significant association between high PSWS and birth weight [*b*= -77.09 (95% CI -121.18– -33.01)] (see supplementary figure S8). No evidence of publication bias was detected (β*_1_*=-0.2, P=0.82).

*Small-for-gestational-age.* Seven studies assessed the relationship between PSWS and SGA (N=113 061) (14, 16, 42, 44, 45, 47, 72). Except for one cross-sectional study (72), all were prospective cohort studies. One study was excluded from the meta-analysis due to a different measurement of the outcome (14). With a very low certainty of evidence, the pooled effect indicated no association between PSWS and SGA [OR 1.15 (95% CI 0.98–1.35)] (see supplementary figure S9). Although publication bias was detected (β*_1_*=3.4, *P*=0.0042), no significant difference was observed between the observed and imputed results [OR 1.022 (95% CI 0.612–1.708)]. Additionally, a stepwise sensitivity analysis indicated that the study done by Tuntiseranee was a potential source of bias (16), but a removal of the study did not change the pooled effect. Hence it was retained in the analysis.

After a thorough qualitative assessment of the study characteristics, a subgroup analysis was conducted to identify potential sources of heterogeneity (*I^2^*=63.36%). The analysis was stratified by sample size, having a sample size ≤5000 (16, 44, 72) [OR 2.22 (95% CI 0.55–8.94)] and having a sample size >5000 (42, 45, 47) [OR 1.09 (95% CI 0.90–1.32)] (see supplementary figure S10). Even though neither subgroup showed statistical significance, the studies having sample sizes ≤5000 demonstrated a stronger association.

## Discussion

The effects of PSWS have been largely overlooked, despite its significance as a critical occupational and public health issue, particularly for pregnant women who are already under considerable physiological stress. The present review is one of the first to synthesize the available empirical data on the associations between PSWS and a range of adverse pregnancy outcomes. The results showed that PSWS had a significant effect on birth weight, pre-eclampsia, PTB, and pregnancy loss. These findings are consistent with previous systematic review results focusing on the physical and environmental aspects of work stress, which examined the effects of shift work, long working hour, prolonged standing, heavy physical work load and occupational noise on adverse pregnancy outcomes (18, 19, 24, 26, 27).

Preeclampsia is a serious complication during pregnancy that poses a threat to mothers’ health and fetal outcomes (73, 74). Our meta-analysis showed that PSWS was associated with an increased risk of pre-eclampsia by approximately 50% with a medium certainty of evidence. This is consistent with other systematic reviews showing that psychological factors increased the risk for pre-eclampsia (36, 75, 76).

A significant but weak association was detected between PSWS and pregnancy loss. This may be due to the included studies in this meta-analysis being limited, and their exposure rates were >10% indicating insufficient statistical power for the analysis. An optimal exposed to non-exposed ratio may be used in future studies to improve the findings.

Preterm birth is another adverse pregnancy outcome that showed a significant 18% increase in occurrence among pregnant women experiencing high PSWS in the present review. Likewise, other review papers also reported significant links between stress and pre-term birth (18, 24, 36). The finding was further stratified by study design, which revealed that only non-prospective studies showed a significant association. This might be due to recall bias and higher exposure rates in cross-sectional and case–control designs as compared to prospective studies.

In addition, we found that PSWS was associated with reduced birth weight with a medium certainty of evidence. Despite the small number of studies, all of them showed strong effects and had good methodological quality, which makes this finding highly reliable and useful for practical applications. In addition, a non-significant association was found between PSWS and LBW. This might be due to high heterogeneity in study designs and sample sizes. In particular, in the same analysis, the sample size of the study that showed a non-significant association (40) was 10 times larger than those in the studies with significant association (14, 15). However, other review studies found significant associations between psychological factors (eg, depression (77) and prenatal stressful conditions (78, 79) and LBW. To conclude, mitigating PSWS among pregnant mothers may be crucial in improving birth weight and avoiding further complications.

In the present review, overall, the associations between PSWS and adverse pregnancy outcomes were rather weak. It is noteworthy that all the studies included in the review were conducted in developed nations, where most enabling factors are readily available, such as regular occupational health checkups and access to other necessary services. The detrimental effects of PSWS on pregnant women in developing countries could be aggravated by the limited availability of these enabling factors and socio-demographic disparities (80–83). According to the ILO report and other evidence, lower and middle-income countries (LMIC) fail to provide flexible maternity leave, relaxed work environments, and screening for pregnant mothers for PSWS by occupational therapists (29). All these factors contribute to a higher prevalence of PSWS (20, 21, 23) and amplify its impact on pregnant mothers. This suggests that the impacts of PSWS might be more concerning in these countries. Usually, in LMIC, attention is often focused on other significant predictors of maternal and child mortality and morbidity, like biomedical determinants, and quality and equity of health care, but overlooked psychosocial risk factors (84, 85). Therefore, investigating the effects of PSWS in LMIC could provide critical insights into its impact on pregnancy outcomes.

Although the link between PSWS and adverse pregnancy outcomes was weak, all included studies adjusted for confounders using multivariable analysis except the Meyer et al study (37). Some also used stratification (38) and matching (17, 37, 48, 67) to minimize residual confounding effects. All these methods strengthen the validity of the observed associations across studies.

PSWS is linked to adverse pregnancy outcomes (pre-eclampsia, pregnancy loss, PTB and LBW) through three primary biological pathways: elevated cortisol levels through the hypothalamus-pituitary-adrenal axis activation, sympathetic nervous system activation, and increased production of pro-inflammatory cytokines (IL-1β and TNF-α) (36, 78, 86, 87). These pathways synergistically or individually affect pregnancy outcomes in three ways. First, an increased production of catecholamines leads to vasoconstriction, endothelial damage, and reduced nitric oxide production, all of which affect blood vessel elasticity. These lead to hypertension in pregnancy, restricting blood flow and hence nutrients to the uterus and placenta, which results in low birth weight and pregnancy loss (36, 78, 86, 87). Second, the disruption of the immune balance leads to inappropriate responses against embryos and disrupts the uterine environment which may result in miscarriage (78, 86). Third, increased production of oxytocin and prostaglandin hormones stimulate cervical ripening and increase uterine contractility that may initiate early labor (36, 78, 86).

PSWS affects pregnancy outcomes not only through biological factors but also a combination of psychological and behavioral responses. Women who are under stress are more engaged in unhealthy behaviors, such as smoking and alcohol consumption, to cope with stress (88–90). This unhealthy coping behavior can further increase the risks of adverse pregnancy outcomes (91–94). Pregnant mothers under stress may also develop depression, anxiety, and loneliness (95–97), all of which are known risk factors for adverse pregnancy outcomes (77, 98–102).

The findings of this review highlight that PSWS may contribute to adverse pregnancy outcomes. Therefore, to mitigate its impacts, employers and managers should consider measures such as reducing workload, flexible working hours and decision-making, flexible maternity leave, and creating a supportive work environment for pregnant women. Clinicians and occupational therapists should pay comparable attention to PSWS screening as other risk screening services during antenatal care. Relevant stakeholders should endorse psychosocial working conditions in occupational health and safety precautions for pregnant mothers. Finally, further research using a robust methodological approach is needed to strengthen or change the direction of the association between PSWS and adverse pregnancy outcomes, especially in LMIC where there has been a lack of evidence so far.

### Study limitations

The authors noted the following limitations: first, all the studies included were from high- and upper-middle-income countries, which makes it difficult to generalize the findings to LMIC. Second, there are methodological concerns, where 84% of the included cohort studies had suboptimal exposed-to-non-exposed ratios, which may compromise the validity and reliability of the results. All the studies were observational, which limits the ability to establish causality. Additionally, some studies assessed JDC with a single question each (45, 50, 69), limiting the JDC model’s components and failing to fully capture its framework. Similarly, Lissåker’s study used a job exposure matrix to evaluate PSWS based on job codes (46), but it was not validated in PSWS evaluation models. Third, the number of studies included in each outcome category was small, which could reduce the statistical power to detect significant effects and may introduce publication bias. Fourth, some studies used administrative data routinely collected by clinics, which has inherent limitations as secondary data (45–47, 50). Fifth, we excluded non-English studies and may have missed important information and introduce publication bias. Sixth, based on the GRADE evaluation of the certainty of evidence for the seven outcomes of interest rated the majority as low or very low. This suggests that the need for more evidence to sufficiently support clinical considerations and policy recommendations. Therefore, given the above-mentioned limitations, the results should be interpreted with caution.

### Concluding remarks

The current review shows that PSWS is evidently associated with pre-eclampsia, pregnancy loss, and preterm birth and reduces infant birth weight. Therefore, it is essential that occupational therapists, employers, and other concerned stakeholders work collaboratively to address and prevent this critical occupational and public health issue.

## Supplementary material

Supplementary material
