# Supplementary material for: The effects of prenatal psychosocial work stress on adverse pregnancy outcomes: A comprehensive systematic review and meta-analysis
Source: Scand J Work Environ Health. 2025 Aug 29;51(5):355–69. doi: 10.5271/sjweh.4236 (PMC12409662; doi:10.5271/sjweh.4236)
Supplement: Supplementary material [file SJWEH-51-355-S001.pdf]

The Effects of Prenatal Psychosocial Work Stress on Adverse Pregnancy Outcomes: A Comprehensive Systematic Review and Meta-analysis[1]

Authors: Wubet Taklual Admas (MPH) [2], Ai Ni Teoh (PhD), Gi Kunchana Chonu (PhD)

1. Supplementary material
2. Correspondence to: Wubet Taklual Admas, Debre Tabor University, College of Health Sciences, Department of Public Health, Ethiopia. [E-mail: wubtak21@gmail.com]

Supplementary file 1

Search strategy form electronic databases.

Search strategy and result using **PubMed** electronic database. The key words search availability in title and abstract.

Number	Search terms	Results until 30 Aug 2024
1.	(((job stress [title/abstract]) or (work-related stress [title/abstract])) or (occupational stress [title/abstract])) or (workplace stress [title/abstract])	8,565
2.	(burnout) or (emotional exhaustion[title/abstract])	30,858
3.	(psychosocial job stress[title/abstract]) or (psychosocial job strain[title/abstract])	52
4.	((((((strain[title/abstract]) or (stress[title/abstract])) or (strenous[title/abstract])) or (demand[title/abstract])) or (control[title/abstract])) or (resource[title/abstract])) and (job[title/abstract])	21,622

5.	(((((occupational[title/abstract]) or (workplace[title/abstract])) or (work related[title/abstract])) or (job[title/abstract])) and (stress[title/abstract])) or (strain[title/abstract])	559,153
6.	1 or2 or 3 or 4 or 5	593,713
7.	(premature birth[title/abstract]) or (preterm birth[title/abstract])	30,805
8.	(premature labor[title/abstract]) or (preterm labor[title/abstract])	9,531
9.	(premature delivery [title/abstract]) or (preterm delivery [title/abstract])	15,711
10.	((((abortion[title/abstract]) or (miscarriages'[title/abstract])) or (fetal loss[title/abstract])) or (still birth[title/abstract])) or (pregnancy loss[title/abstract])	78,747
11.	(((birth weight[title/abstract]) or (low birth weight[title/abstract])) or (small for gestational age[title/abstract])) or (fetal growth[title/abstract])	96,237
12.	((gestational hypertension[title/abstract]) or (pregnancy induced hypertension[title/abstract])) or (hypertensive disorder of pregnancy[title/abstract])	9,436
13.	(hypertension[title/abstract]) and (pregnancy[title/abstract])	24,024
14.	(gestational diabetic mellitus[title/abstract]) or (pregnancy induced diabetic mellitus[title/abstract])	22
15.	(diabetic mellitus[title/abstract]) and (pregnancy[title/abstract])	26
16.	gestational diabetic mellitus[title/abstract]	18

17.	(pregnancy outcomes[title/abstract]) or (pregnancy complications[title/abstract])	43,714
18.	(adverse pregnancy outcomes[title/abstract]) or (adverse birth outcomes[title/abstract])	10,382
19.	7 or 8 or 9 or 10 or 11	205,324
20.	12 or 13 or 14 or 15 or 16	25,121
21.	17 or 18	45,756
22.	6 and 19	1,632
23.	6 and 20	131
24.	6 and 21	186
25.	22 or 23 or 24	1,837
26.	Restricted by English language and human species	786

Search strategy and result using **PsycINFO** electronic database. The key words search availability in title and abstract (tiab=Title and abstract).

**Number**	**Search terms**	**Results until 02 Sep 2024**
S1	tiab(Job stress) OR tiab(Work related stress) OR tiab(Occupational stress) OR tiab(Work place stress) OR (Work stress)	69,785
S2	tiab(Burnout) OR tiab(Fatigue) OR tiab(Emotional exhaustion)	47,743
S3	tiab(Psychological Job Stress) OR tiab(Psychological Job strain)	2,676

S4	tiab(Occupational) OR tiab(Work place) OR tiab(Work related) OR tiab(Work) AND strain	163,775
S5	tiab(Occupational) OR tiab(Work place) OR tiab(Work related) OR tiab(Work) AND tiab(strain)	162,969
S6	tiab("Job strain") OR tiab("Strenuous Job ") OR tiab("Work demand") OR tiab("Job demand ") OR tiab("Job control") OR tiab("Work control") OR tiab("Job demand-resource") OR tiab("Effort reward")	3,162
S7	[S1] OR [S2] OR [S3] OR [S4] OR [S5] OR [S6]	246,079
S8	tiab("Preterm") OR tiab("Premature ") AND tiab(Birth) OR tiab(infant) OR tiab(Delivery) OR tiab(Labor)	176,189
59	tiab(Abortion) OR tiab(Miscarriage's) OR tiab(fetal loss) OR tiab(stillbirth) OR tiab(pregnancy loss) OR tiab(still-birth)	8,841
S10	tiab(birth weight) OR tiab(low birth weight) OR tiab(small for gestational age) OR tiab(fetal growth)	9,802
S11	tiab(Hypertensive disorder of pregnancy) OR tiab(Gestational hypertension) OR tiab(Pregnancy induced hypertension)	483
S12	tiab(Hypertension) AND tiab(Pregnancy)	572
S13	tiab(gestational diabetic mellitus) OR tiab(Pregnancy induced Diabetic Mellitus)	26

S14	tiab(Diabetic Mellitus) AND tiab(Pregnancy)	30
S15	tiab("pregnancy Outcomes") OR tiab("Pregnancy complications")	1,735
S16	tiab(Adverse birth outcomes) OR tiab(Adverse pregnancy outcomes)	3,338
517	S8 or S9 0r S10 or	188,010
S18	S11 OR S12 OR S13 OR S14	779
S19	S15 OR S16	4,483
S20	S7 AND S17	11,986
S21	S7 AND S18	26
S22	S7 AND S19	199
S23	S20 or S21 OR S22	12,100
524	Limited by language and pregnancy	555

Search strategy and result using **Scopus** electronic database. Key words availability in Title, Abstract, and keywords (TITLE-ABS-KEY= Title, abstract, and Keywords).

**Number**	**Search terms**	**Result until 05 Sep 2024**
1.	(TITLE-ABS-KEY (“Job stress”) OR TITLE-ABS-KEY (“Work related stress”) OR TITLE-ABS-KEY ("Work stress") OR TITLE-ABS-KEY ("Occupational stress") OR TITLE-ABS-KEY ("Work place stress"))	29,363

2.	(TITLE-ABS-KEY (“Job”) OR TITLE-ABS-KEY ("Work related ") OR TITLE-ABS-KEY ("Work") OR TITLE-ABS-KEY ("Occupational ") OR TITLE-ABS-KEY ("Work place") AND TITLE-ABS-KEY ("Strain"))	188,486
3.	(TITLE-ABS-KEY ("Psychosocial Job stress") OR TITLE-ABS-KEY ("Psychosocial Job strain") OR TITLE-ABS-KEY ("Psychosocial work stress") OR TITLE-ABS-KEY ("Psychosocial work strain"))	139
4.	(TITLE-ABS-KEY ("Job") AND TITLE-ABS-KEY ("Control") OR TITLE-ABS-KEY ("Resource") OR TITLE-ABS-KEY ("Strenuous") OR TITLE-ABS-KEY ("Demand"))	130,702
5.	(TITLE-ABS-KEY ("Burnout") OR TITLE-ABS-KEY ("Fatigue") OR TITLE-ABS-KEY ("Emotional exhaustion") AND TITLE-ABS-KEY (job) OR TITLE-ABS-KEY (work))	89,225
6.	TITLE-ABS-KEY ("Psychosocial factor")	424,981
7.	1 or 2 or 3 or 4 or 5 or 6	
8.	(TITLE-ABS-KEY ("Preterm") OR TITLE-ABS-KEY ("Premature") OR TITLE-ABS-KEY ("Pre-term") AND TITLE-ABS-KEY (birth) OR TITLE-ABS-	214,381

	KEY (labor) OR TITLE-ABS-KEY (delivery) OR TITLE-ABS-KEY (infant))	
9.	(TITLE-ABS-KEY ("Abortion") OR TITLE-ABS-KEY ("Miscarrages") OR TITLE-ABS-KEY ("Stillbirth") OR TITLE-ABS-KEY ("Still-birth") OR TITLE-ABS-KEY ("Fetal loss") OR TITLE-ABS-KEY ("Pregnancy loss"))	188,543
10.	(TITLE-ABS-KEY ("Birth weight") OR TITLE-ABS-KEY ("Low birth weight") OR TITLE-ABS-KEY ("Small for gestational age") OR TITLE-ABS-KEY ("Fetal growth"))	191,498
11.	8 or 9 or 10	499,096
12.	(TITLE-ABS-KEY ("Gestational hypertension") OR TITLE-ABS-KEY ("Pregnancy induced hypertension") OR TITLE-ABS-KEY ("Hypertensive disorder of pregnancy"))	14,009
13.	(TITLE-ABS-KEY ("Hypertension") AND TITLE-ABS-KEY ("Pregnancy"))	62,887
14.	(TITLE-ABS-KEY ("Diabetic Mellitus") AND TITLE-ABS-KEY ("Pregnancy"))	61
15.	(TITLE-ABS-KEY ("Gestational Diabetic Mellitus") OR TITLE-ABS-KEY ("Pregnancy induced Diabetic mellitus") OR TITLE-ABS-KEY ("Pregnancy in Diabetic") OR TITLE-ABS-KEY ("Pregnancy in Diabetes") OR TITLE-ABS-KEY ("Gestational, Diabetes"))	38,858

16.	12 or 13 or 14 or 15	95,783
17.	(TITLE-ABS-KEY ("Pregnancy outcomes ") OR TITLE-ABS-KEY ("Outcomes,Pregnancy") OR TITLE-ABS-KEY ("Pregnancy complications"))	258,603
18.	(TITLE-ABS-KEY (" Adverse birth outcomes ") OR TITLE-ABS-KEY ("Adverse pregnancy outcomes"))	13,313
19.	17 or 18	259,450
20.	7 and 11	1,350
21.	7 and 16	211
22.	7 and 19	727
23.	20 or 21 or 22	1,876
24.	Limited by articles only and English language and exclude Animal studies	1213

Search strategy and result using **CINHAL** electronic databases. Key words availability in Abstract (AB=Abstract)

**Number**	**Search terms**	**Result until 5 Sep 2024**
1.	AB Job stress OR AB Work related stress OR AB Occupational stress OR AB Work stress OR AB Workplace stress	8,036
2.	AB Occupational OR AB Workplace OR AB Work related OR AB Work OR AB Job AND AB Strain	187,044

3.	AB "Job" AND AB "Demand" OR AB "Strenuous" OR AB "Control" OR AB "Resource"	383,543
4.	AB "Psychosocial Job stress" OR AB "Psychosocial Job strain" OR AB "Psychosocial work stress" OR AB "Psychosocial Work strain"	26
5.	AB "Psychosocial factors" OR AB "Psychosocial effects" OR AB "Psychosocial stressors"	2,433
6.	AB "Burnout" OR AB "Fatigue" OR AB "Exhaustion"	20,469
7.	S1 OR S2 OR S3 OR S4 OR S5 OR S6	563,261
8.	AB Preterm OR AB Pre-term OR AB Premature AND AB Birth OR AB Labor OR AB Delivery OR AB Infant	242,254
9.	AB Abortion OR AB miscarriage OR AB "Fetal loss" OR AB "Pregnancy loss" OR AB "Stillbirth" OR AB "Still-birth" OR AB "stillborn" OR AB "intrauterine death" OR AB "Intrauterine fetal death"	18,654
10.	AB "Birth weight" OR AB "Birth size" OR AB "Low birth weight" OR AB "Small for gestational age" OR AB fetal growth	26,711
11.	AB "hypertensive disorder of pregnancy OR AB "preeclampsia" OR AB "Eclampsia" OR AB "Pre-clampsia" OR AB "Gestational hypertension" OR AB "Pregnancy induced hypertension"	12,556
12.	AB "Gestational Diabetes" OR AB "Gestational Diabetes Mellitus" OR AB "Diabetes, Pregnancy" OR AB "Pregnancy induced Diabetes"	7,403

13.	AB "Diabetes Mellitus" AND AB "Pregnancy" OR AB "Gestational"	42,034
14.	AB "Pregnancy outcomes" OR AB "Pregnancy complications" OR AB "Adverse pregnancy outcomes" OR "Adverse birth outcomes"	9,510
15.	S8 OR S9 OR S10	262,084
16.	S11 OR S12 OR S13	49,333
17.	S7 AND S14	388
18.	S7 AND S15	2,151
19.	S7 AND S16	1,268
20.	S17 OR S18 OR S19 restricted by English language and exclude Medline outputs	2,719

Search strategy and result using **Science Citation index (SCI) expanded** electronic databases. Keywords availability in Title, abstract, and key words (TS=key word availability in Title, abstract and keywords)

Number	Search terms	Result until 24 Sep 2024
1.	(((((TS=("Job stress")) OR TS=("Work stress")) OR TS=("Occupational stress")) OR TS=("Work related stress")) OR TS=("Work-related stress")) OR TS=("Workplace stress")	17,724
2.	"Job strain " (Topic) or "Work strain" (Topic) or "Occupational strain" (Topic) or "Workplace Strain" (Topic) or "Work related strain" (Topic) or Work-related strain (Topic)	3,690

3.	"Job strenuous " (Topic) or "Job demand " (Topic) or "Job control" (Topic) or "Jab demand-resource" (Topic) or "Job demand-control" (Topic) or "Strenuous work" (Topic) or "Demanding Work" (Topic) or "Work control" (Topic)	2,569
4.	"Burnout " (Topic) or "Fatigue " (Topic) or "Exhaustion" (Topic)	318,883
5.	"Psychosocial Job stress " (Topic) or "Psychosocial Job strain " (Topic)	55
6.	"Stress" (Topic) or "Psychosocial factors" (Topic)	15,558
7.	#1 OR #2 OR #3 OR #4 OR #5 OR #6	342,627
8.	"Premature birth" (Topic) or "Premature baby" (Topic) or "Premature infant" (Topic)	9,602
9.	"Preterm baby" (Topic) or "Preterm birth" (Topic) or "Preterm infant" (Topic) and "Preterm delivery" (Topic) or "Preterm labor " (Topic)	38,263
10.	"Abortion" (Topic) or "Miscarriage's" (Topic) or "Fetal loss" (Topic) or "Pregnancy loss" (Topic) or "stillbirth " (Topic) or "still-birth " (Topic) or "Intrauterine death" (Topic) or "Intra-uterine death" (Topic)	72,104
11.	"Birth weight" (Topic) or "low birth weight" (Topic) or "Small for gestational age" (Topic) or "fetal growth" (Topic)	118,752
12.	#8 OR #9 OR #10 OR #11	215,297
13.	"Gestational hypertension" (Topic) or "Pregnancy induced hypertension" (Topic) or "Hypertensive disorder of	54,715

	pregnancy" (Topic) or "Preeclampsia" (Topic) or "Pre-eclampsia " (Topic) or "Eclampsia " (Topic)	
14.	"Gestational Diabetes" (Topic) or "Gestational Diabetes Mellitus" (Topic) or "Pregnancy induced Diabetes" (Topic) or "Pregnancy induced Diabetes Mellitus" (Topic) or "Diabetes, Pregnancy" (All Fields)	25,955
15.	#13 OR #14	76,469
16.	"Pregnancy outcomes" (Topic) or "Pregnancy complications" (Topic) or "Adverse pregnancy outcomes" (Topic) or "Adverse birth outcomes" (Topic)	36,160
17.	#7 AND #12	688
18.	#7 AND #15	190
19.	#7 AND #16	191
20.	#17 OR #18 OR #19	900
21.	Restricted #20 by research articles	732
22.	https://www.webofscience.com/wos/woscc/summary/8fe5b28e-af37-4158-b3a2-062030ea533b-01047fc1d9/relevance/1	

Search strategy and result using **Cochrane** electronic databases. Keywords availability in Title, abstract, and key words (ti= title, ab= abstract, kw= key word).

**Number**	**Search terms**	**Result until 30 Nov 2024**

1.	(Job strain):ti,ab,kw OR (Work strain):ti,ab,kw OR (Occupational strain):ti,ab,kw OR (Work related strain):ti,ab,kw OR (work place strain):ti,ab,kw	1417
2.	(“Job stress"):ti,ab,kw OR ("Work stress"):ti,ab,kw OR ("Occupational stress"):ti,ab,kw OR ("Work related stress"):ti,ab,kw OR ("work place stress"):ti,ab,kw	1153
3.	("Job strenuous "):ti,ab,kw OR ("Job demand "):ti,ab,kw OR ("Job control"):ti,ab,kw OR ("Job demand-resource"):ti,ab,kw OR ("Job demand-control"):ti,ab,kw	144
4.	("Burnout "):ti,ab,kw OR ("Fatigue "):ti,ab,kw OR ("Exhaustion"):ti,ab,kw	56591
5.	("Psychosocial Job stress "):ti,ab,kw OR ("Psychosocial job strain ") :ti,ab,kw	1
6.	("Stress"):ti,ab,kw OR ("Psychosocial factors"):ti,ab,kw	93556
7.	#1 OR #2 OR #3 OR #4 OR #5 OR #6	145290
8.	("Premature birth"):ti,ab,kw OR ("Premature baby"):ti,ab,kw OR ("Premature infant"):ti,ab,kw	7405
9.	("Preterm baby"):ti,ab,kw OR ("Preterm birth"):ti,ab,kw OR ("Preterm infant"):ti,ab,kw OR ("Preterm delivery"):ti,ab,kw OR ("Preterm labor "):ti,ab,kw	14668
10.	("Abortion"):ti,ab,kw OR ("Miscarriage's"):ti,ab,kw OR ("Fetal loss"):ti,ab,kw OR ("stillbirth "):ti,ab,kw OR ("Intrauterine death"):ti,ab,kw	13475

11.	("Birth weight"):ti,ab,kw OR ("low birth weight"):ti,ab,kw OR ("Small for gestational age"):ti,ab,kw OR ("fetal growth"):ti,ab,kw	15486
12.	#8 OR #9 OR #10 OR #11	40001
13.	("Gestational hypertension"):ti,ab,kw OR ("Pregnancy induced hypertension"):ti,ab,kw OR ("Hypertensive disorder of pregnancy"):ti,ab,kw OR ("Preeclampsia"):ti,ab,kw AND ("Eclampsia "):ti,ab,kw	3978
14.	("Gestational Diabetes"):ti,ab,kw OR ("Gestational Diabetes Mellitus"):ti,ab,kw OR ("Pregnancy induced Diabetes"):ti,ab,kw OR ("Pregnancy induced Diabetes Mellitus"):ti,ab,kw AND ("Diabetes, Pregnancy"):ti,ab,kw	3977
15.	#13 OR #14	7403
16.	("Pregnancy outcomes"):ti,ab,kw OR ("Pregnancy complications"):ti,ab,kw OR ("Adverse pregnancy outcomes"):ti,ab,kw OR ("Adverse birth outcomes"):ti,ab,kw	13909
17.	#7 AND #12	638
18.	#7 AND #15	430
19.	#7 AND #16	748
20.	#17 OR #18 OR #19	1573
21.	https://www.cochranelibrary.com/advanced-search/search-manager	

Supplementary file 2. Methodological quality assessment of included studies using the JBI criteria specific to study design

Table: 2.1. Risk of bias assessment for cross-sectional studies

References	Were the criteria for inclusion in the sample	Were the study subjects and the setting described in detail?	Was the exposure measured in a valid and reliable way?	Were objective, standard criteria used for measurement of the condition?	Were confounding factors identified?	Were strategies to deal with confounding factors stated?	Were the outcomes measured in a valid and reliable way?	Was appropriate statistical analysis used?	Overall Risk of bias
Ceron-Mireles et al. (1996)	yes	Yes	Yes	Yes	Yes	Yes	Yes	Yes	Low risk
Meyer et al (2007)	no	Yes	yes	yes	yes	yes	Yes	yes	Low risk

Table:2.2. Risk of bias assessment for case-control studies

Reference	Were the groups comparable other than the presence of disease	Were cases and controls matched appropriately?	Were the same criteria used for identification of cases and controls?	Was exposure measured in a standard, valid and reliable way?	Was exposure measured Similarly	Were confounding factors identified?	Were strategies to deal with confounding factors stated?	Were outcomes assessed in a standard way	Was the exposure period of interest long enough?	Was appropriate statistical analysis used?	Overall risk of bias
Brandt et al. (1992),	No	No	Yes	yes	yes	yes	yes	no	Yes	Yes	Medium risk
Breet et al. (1997), USA	No	no	Yes	yes	yes	yes	yes	yes	Yes	yes	Low risk

Croteau et al. (2007)	No	No	Yes	yes	yes	yes	yes	yes	Yes	yes	Low risk
Haelterman et al. (2007),	No	no	No	yes	yes	yes	yes	yes	Yes	yes	Medium risk
Marcoux et al. (1999),	no	no	Yes	yes	yes	yes	yes	yes	Yes	yes	Low risk
Escriba-Agüir et al. (2001)	no	no	No	yes	yes	yes	yes	yes	Yes	yes	Low risk

Table: 2.3. Risk of bias assessment for Cohort studies

References	Were the two groups similar and recruited from the same population?	Were the exposures measured similarly in both group	Was the exposure measured in a valid and reliable	Were confounding factors identified?	Were strategies to deal with confounding factors stated?	Were the groups free of the outcome at the start of the study	Were the outcomes measured in a valid and reliable way?	Was the follow up time reported and sufficient to be long enough for outcomes	Was follow up complete, and if not, were the reasons to loss to follow up	Were strategies to address incomplete follow up utilized?	Was appropriate statistical analysis used?	Overall risk of bias
Fenster et al. (1995),	no	yes	yes	yes	yes	yes	yes	yes	yes	yes	yes	Low risk
Henriksen et al. (1994),	no	yes	yes	yes	yes	yes	yes	yes	no	yes	yes	Low risk
Homer et al. (1990)	no	yes	yes	yes	yes	yes	yes	yes	no	yes	yes	Low risk
Klonoff-Cohen et al. (1996)	yes	yes	yes	yes	yes	yes	yes	yes	yes	yes	yes	Low risk

Landsbergis et al. (1996)	no	yes	yes	yes	yes	yes	yes	yes	yes	yes	yes	Low risk
Larsen et al. (2013)	no	yes	yes	yes	yes	yes	yes	yes	yes	no	yes	Low risk
Larsen et al. (2014)	no	yes	yes	yes	yes	yes	yes	yes	no	no	yes	Medium Risk
Lee et al. (2011).	no	yes	yes	yes	yes	yes	no	yes	no	no	no	Medium Risk
Lissåker et al. (2022)	no	yes	yes	yes	yes	yes	yes	yes	no	no	yes	Medium Risk
Meyer et al (2016)	yes	yes	yes	yes	yes	yes	no	yes	yes	no	no	Medium Risk
Meyer et al (2017)	yes	yes	yes	yes	yes	yes	no	yes	yes	no	no	Medium Risk
Oths, et al. (2001),	no	yes	yes	yes	yes	yes	yes	yes	yes	yes	no	Low risk
Sejbaek etal. (2018),	no	yes	yes	yes	yes	yes	yes	yes	yes	no	yes	Low risk
Tuntiseranee et al. (1998).	no	yes	yes	yes	yes	yes	yes	yes	no	no	no	Medium risk
Vollebregt et al (2007),	no	yes	yes	yes	yes	yes	yes	yes	yes	yes	yes	Low risk
Vrijkotte et al. (2021),	no	yes	yes	yes	yes	yes	yes	yes	yes	yes	yes	Low risk
Vrijkotte et al. (2009),	no	yes	yes	yes	yes	yes	yes	yes	yes	yes	yes	Low risk
Zhu, et al. (2004),	no	yes	yes	yes	yes	yes	yes	yes	yes	no	yes	Low risk

Supplementary file 3. Certainty assessment of using GRADE

The overall evidence assessment between Psychosocial work stress and Adverse pregnancy outcomes									
The association between Psychosocial Work stress and Gestational Hypertension									
No of studies	Study design	Risk of bias	Inconsistency	Indirectness of evidence	Imprecision	Publication bias	Effect (Cofounding, Effect size, large magnitude of effect)	Certainty of evidence	Strength of message
(n=5)	Observational studies	Not series	Not concern	Not concern	Not Serious	Not serious	No reason to rate up	Low	"Not enough evidence from the scientific literature to guide policymakers, clinicians, and patients"
The association between Psychosocial Work stress and Pre-eclampsia									
No of studies	Study design	Risk of bias	Inconsistency	Indirectness of evidence	Imprecision	Publication bias	Effect	Certainty of evidence	Strength of message
(n=5)	Observational studies	Not serious	Not concern	Not concern	Not concern	Not serious	Rate up due to controlling residual confounder	Moderate	"Practice considerations" (moderate)
The association between Psychosocial Work stress and pregnancy loss									
No of studies	Study design	Risk of bias	Inconsistency	Indirectness of evidence	Imprecision	Publication bias	Effect	Certainty of evidence	Strength of message
(n=4)	Observational studies	Not serious	Not concern	Not concern	No concern	Not serious	No reason to rate up	low	"Not enough evidence from the scientific literature to guide policymakers, clinicians, and patients"
The association between Psychosocial Work stress and preterm birth									
No of studies	Study design	Risk of bias	Inconsistency	Indirectness of evidence	Imprecision	Publication bias	Effect	Certainty of evidence	Strength of message
(n=9)	Observational studies	Not serious	Not concern	Not concern	No concern	Not serious	Rate up due to contracting residual confounders	Moderate	"Practice considerations" (moderate)
The association between Psychosocial Work stress and low birth weight									

No of studies	Study design	Risk of bias	Inconsistency	Indirectness of evidence	Imprecision	Publication bias	Effect	Certainty of evidence	Strength of message
(n=3)	Observational studies	Not serious	serious	Not serious	serious	serious	No reason to rate up	Very low	“Not enough evidence from the scientific literature to guide policymakers, clinicians, and patients”
The association between Psychosocial Work stress and birth weight									
No of studies	Study design	Risk of bias	Inconsistency	Indirectness of evidence	Imprecision	Publication bias	Effect	Certainty of evidence	Strength of message
(n=4)	Observational studies	Not serious	Not serious	Not serious	Not serious	Not serious	Rate up due to high magnitude of effect size	Medium	“Practice considerations”
The association between Psychosocial Work stress and Small for gestational age									
No of studies	Study design	Risk of bias	Inconsistency	Indirectness of evidence	Imprecision	Publication bias	Effect	Certainty of evidence	Strength of message
(n=6)	Observational studies	Not serious	Serious	Not serious	Not serious	Serious	No reason to rate up	Very low	“Not enough evidence from the scientific literature to guide policymakers, clinicians, and patients”

Supplementary file 4. Forest plots of each outcome.

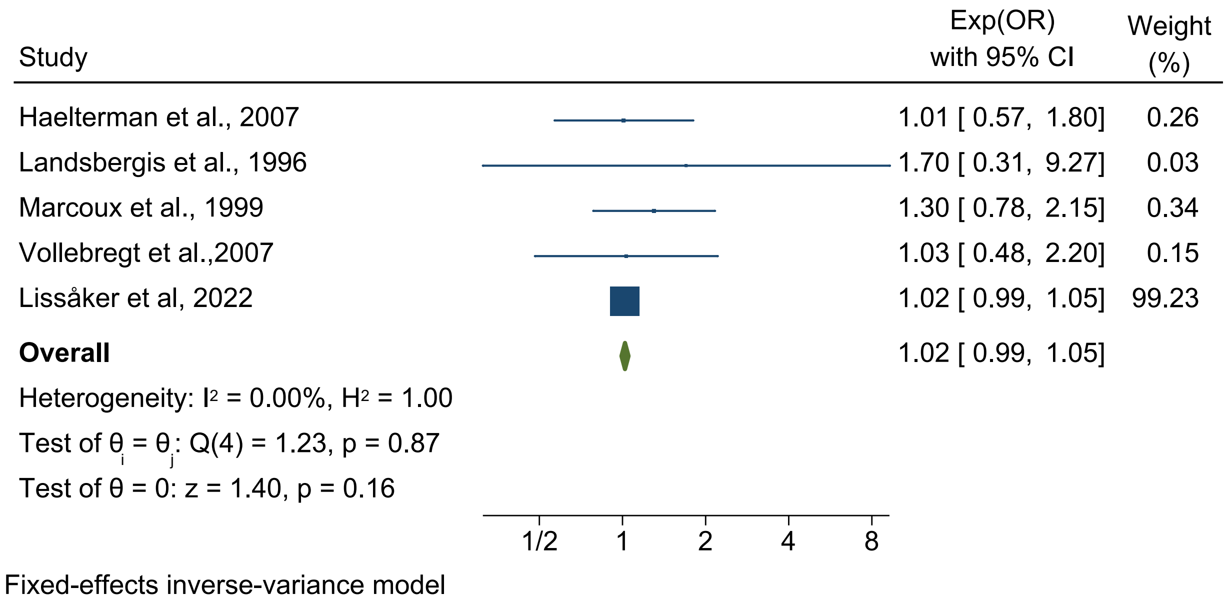

Figure S1: The forest plot for psychosocial work stress and hypertensive disorder of pregnancy

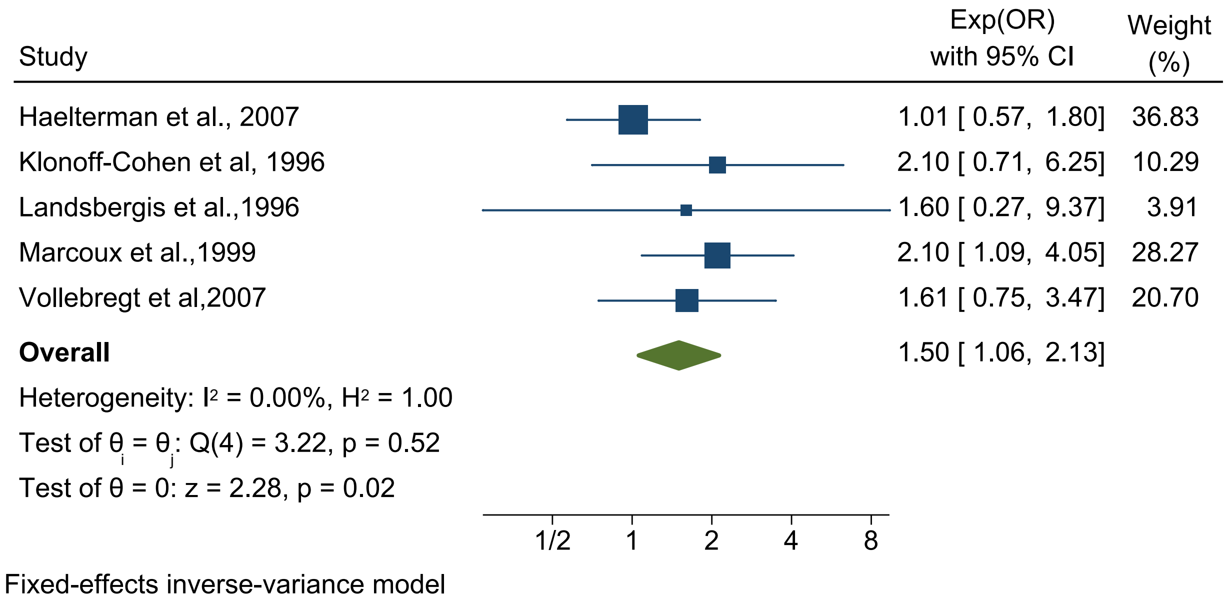

Figure S2: The forest plot for psychosocial work stress and pre-eclampsia

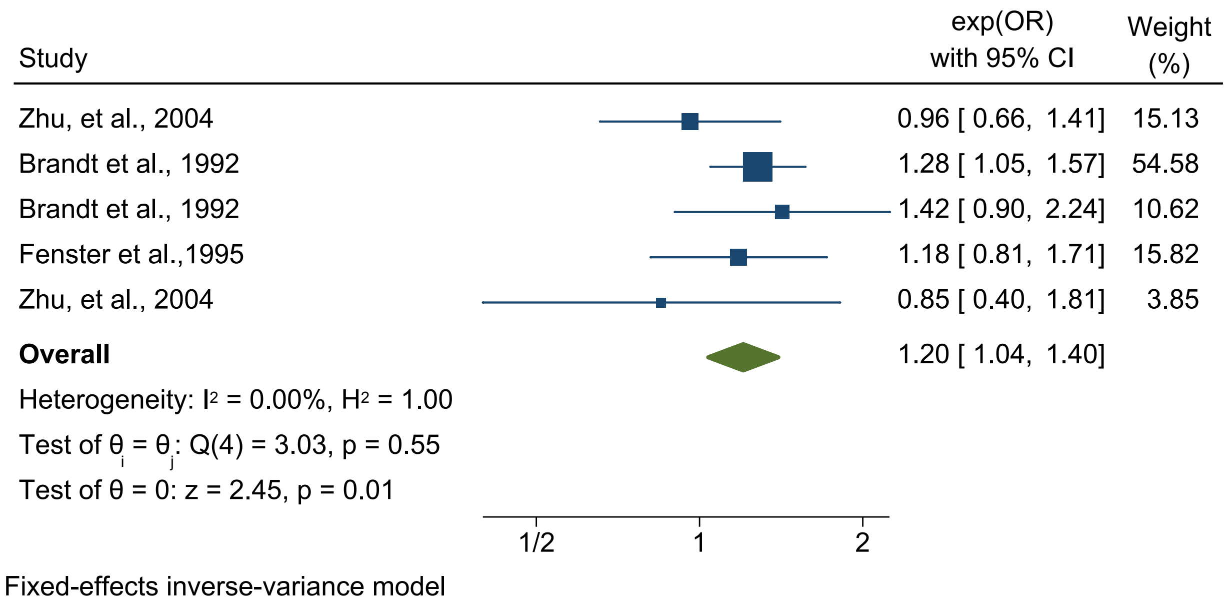

Figure S3: The forest plot for psychosocial work stress and pregnancy loss

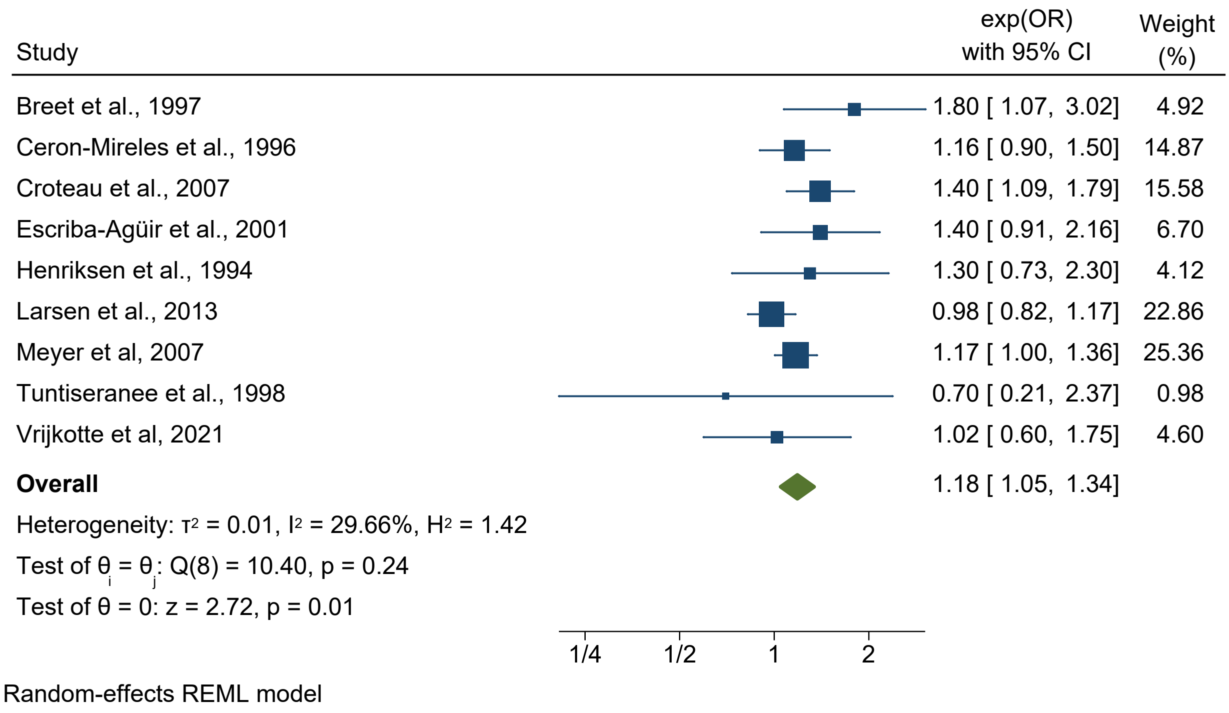

Figure S4: The forest plot for psychosocial work stress and preterm birth

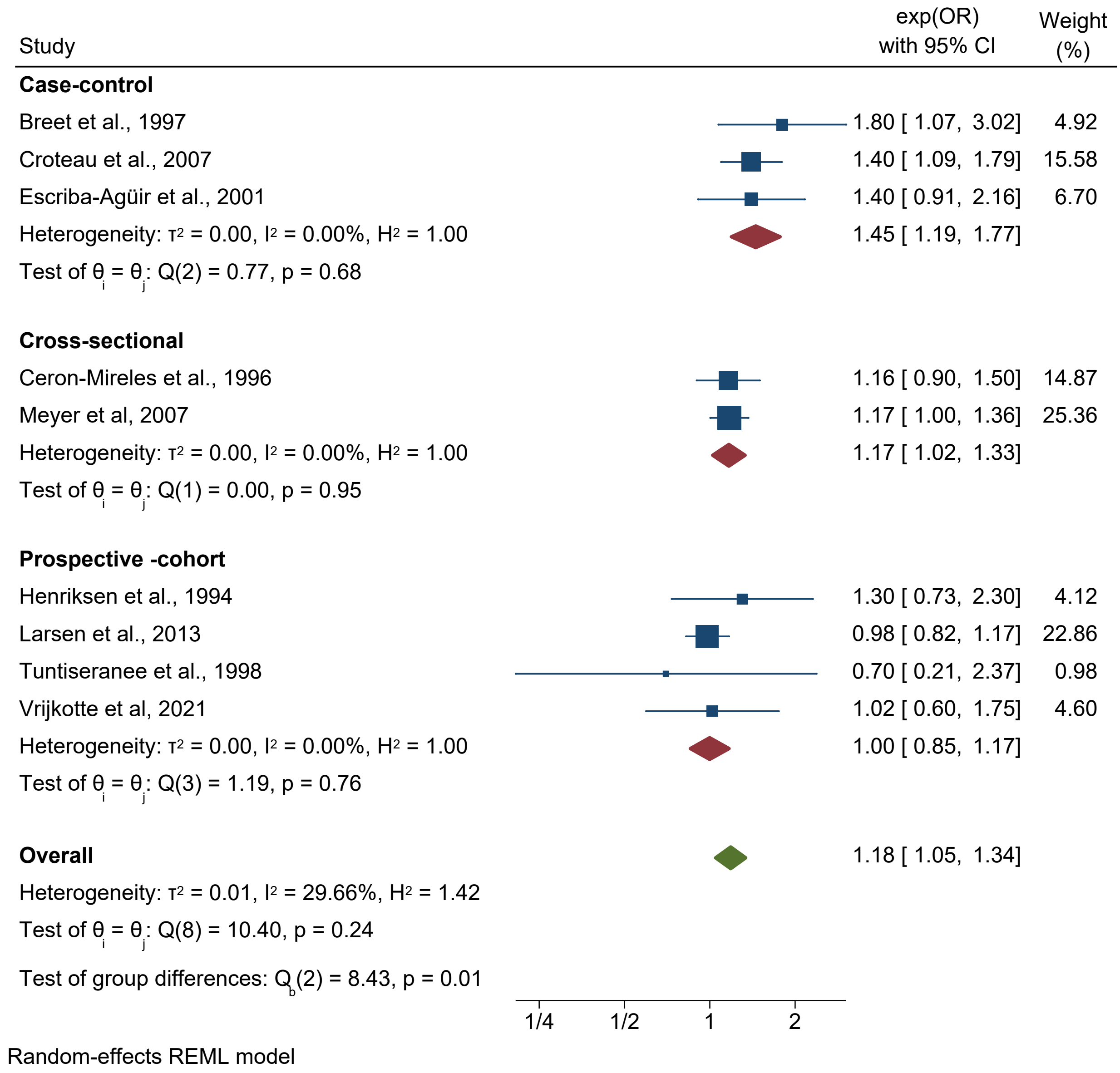

Figure S5: The forest plot for PSWS and PTB stratified by study design.

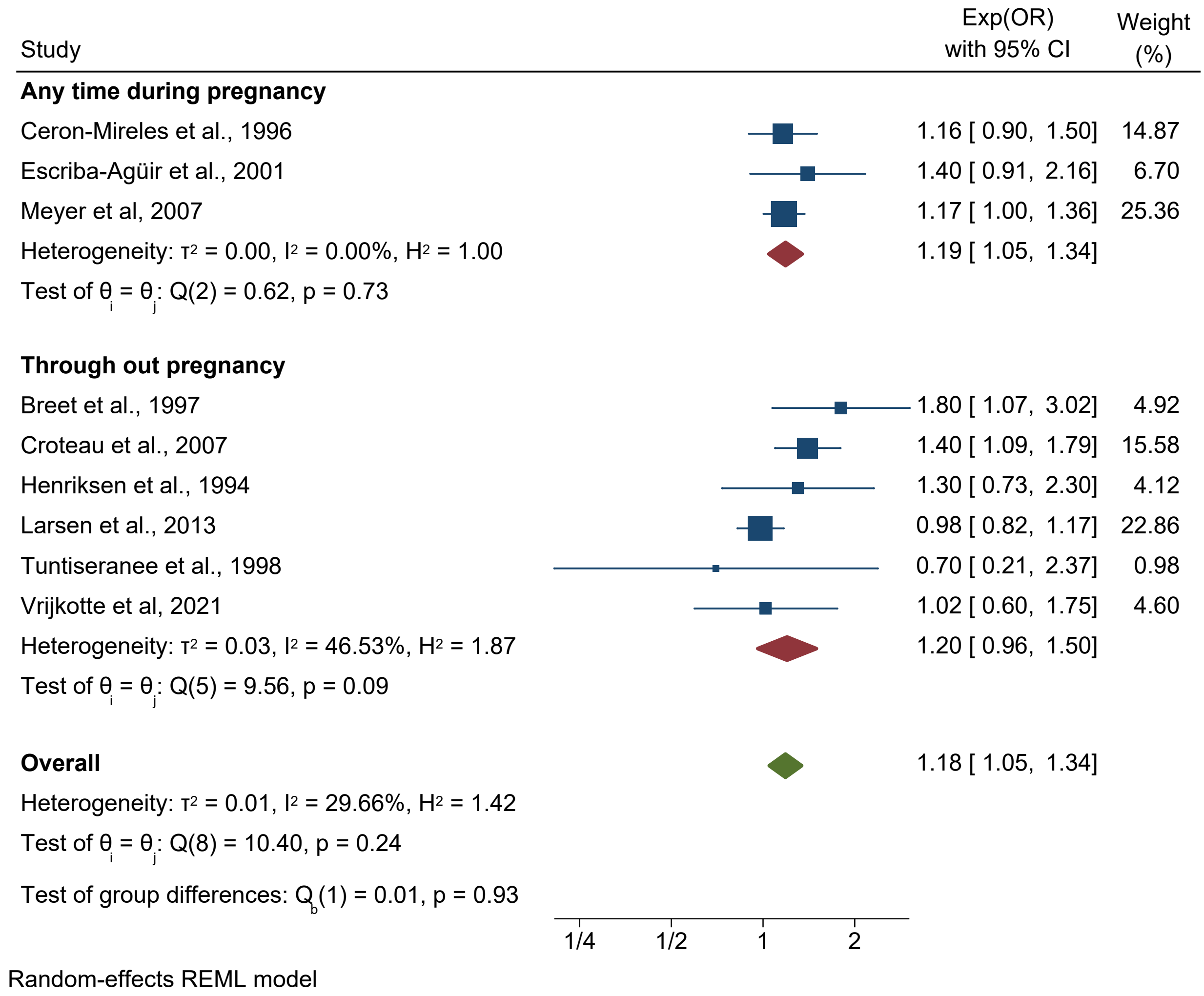

Fig S6: The forest plot for psychosocial work stress and preterm birth stratified by exposure during pregnancy.

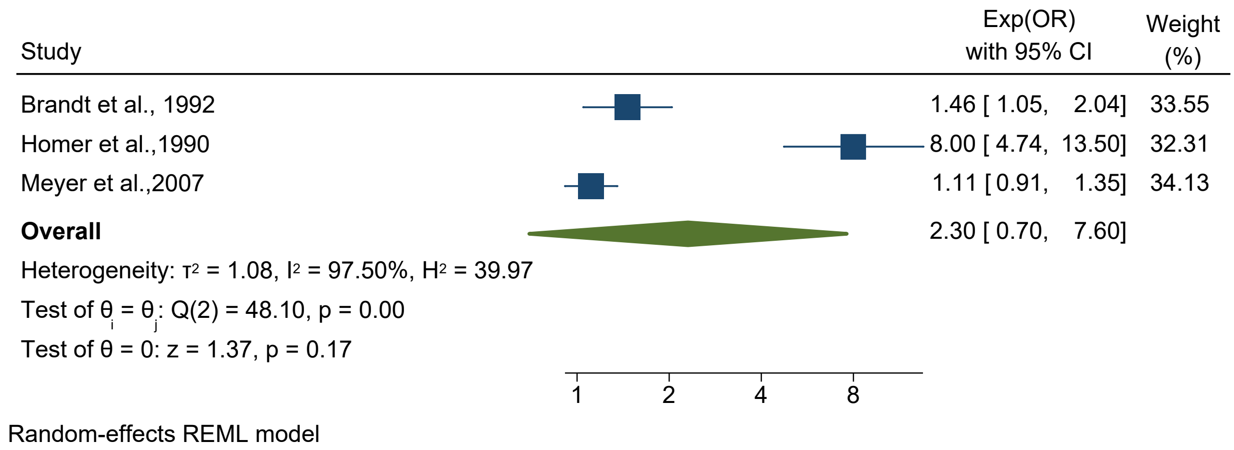

Figure S7: The forest plot for psychosocial work stress and low birth weight

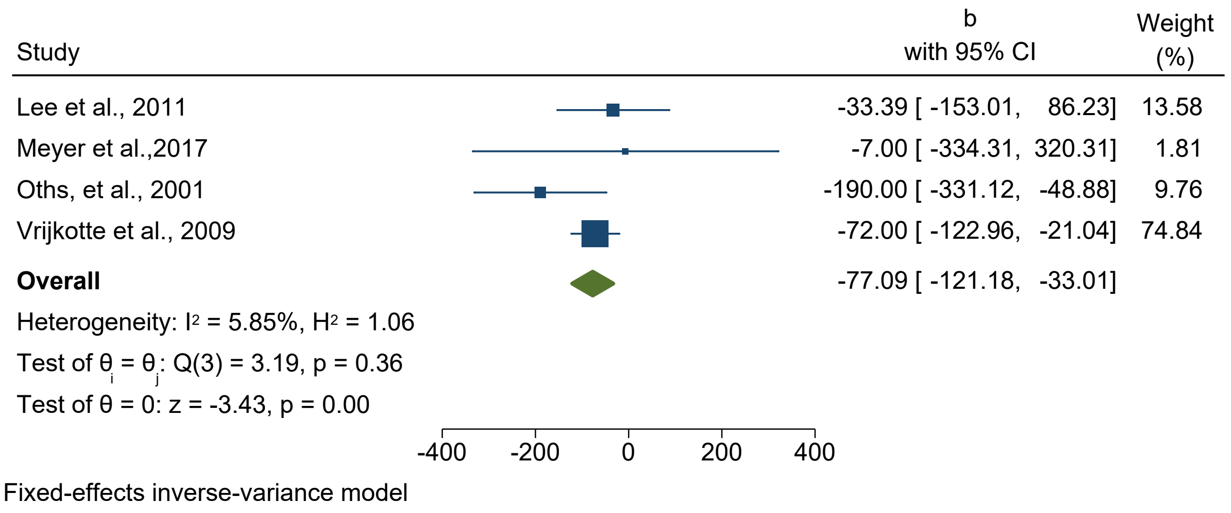

Figure S8: The forest plot for psychosocial work stress and birth weight

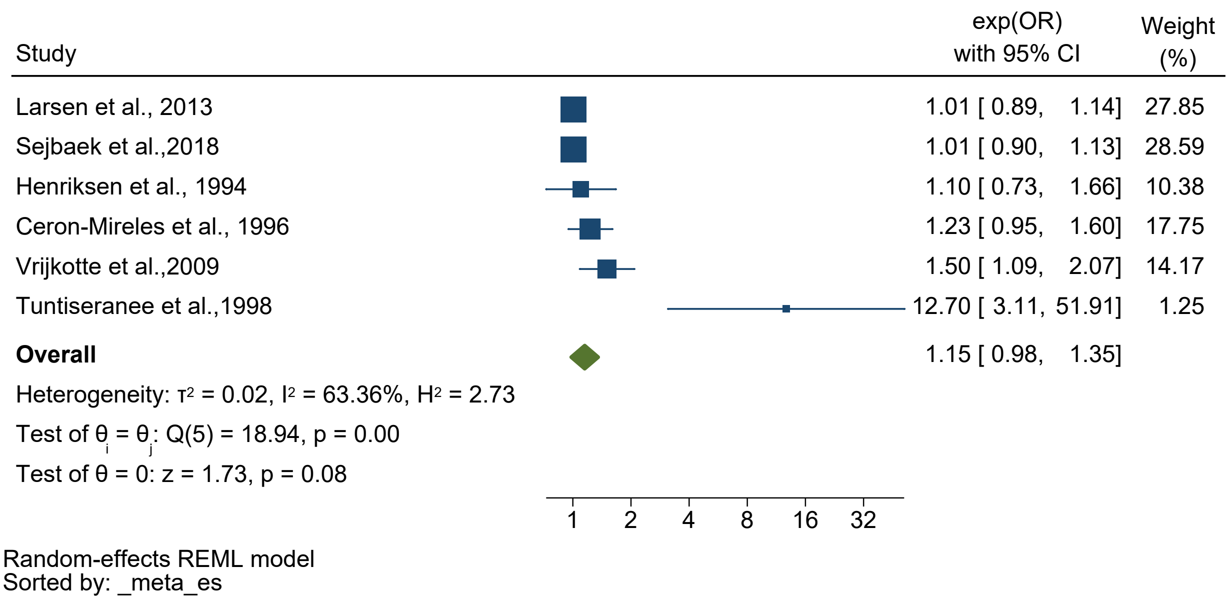

Figure S9: The forest plot for psychosocial work stress and small for gestational age

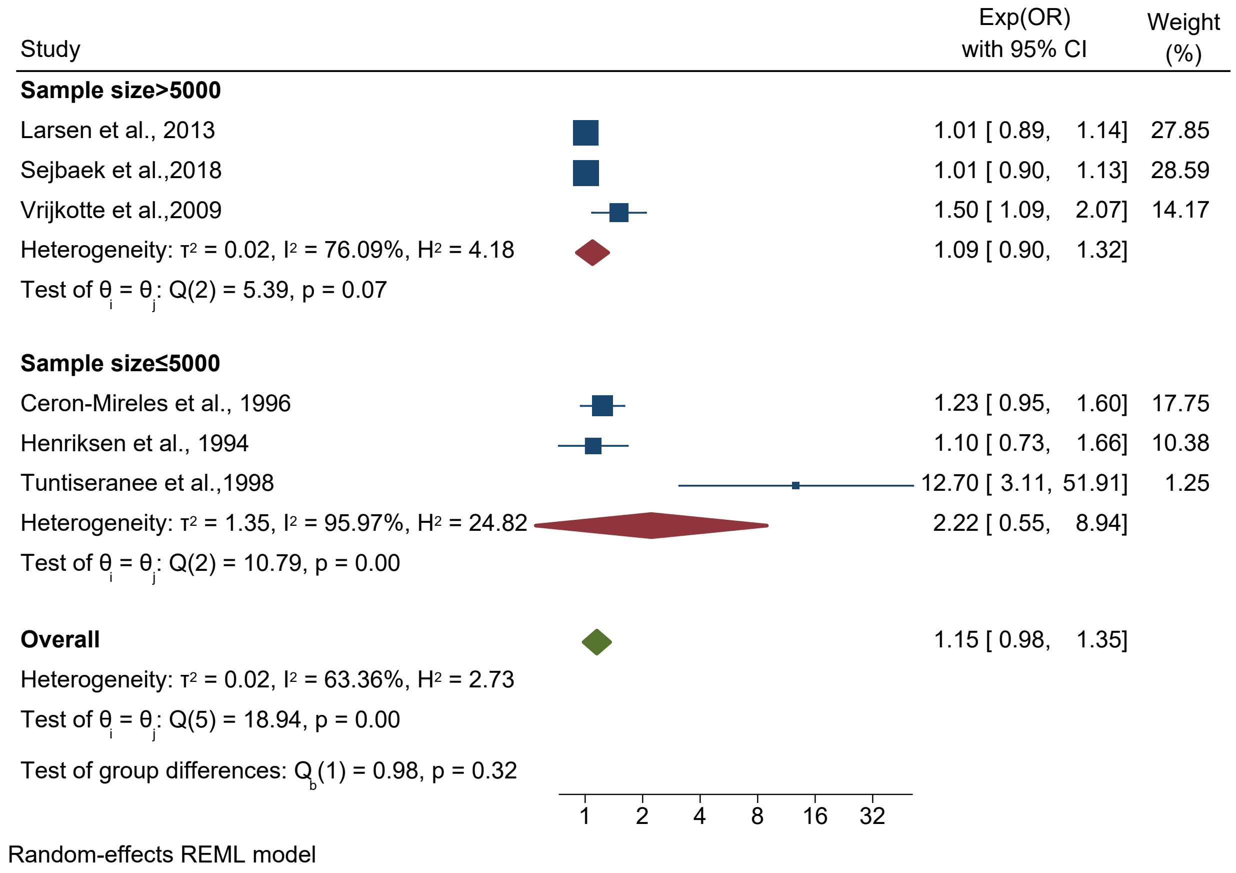

Figure S10: The forest plot for psychosocial work stress and small for gestational age stratified by sample size
